# A non-enzymatic glucose sensor enabled by bioelectronic pH control

**DOI:** 10.1038/s41598-019-46302-9

**Published:** 2019-07-26

**Authors:** Xenofon Strakosas, John Selberg, Pattawong Pansodtee, Nebyu Yonas, Pattawut Manapongpun, Mircea Teodorescu, Marco Rolandi

**Affiliations:** Department of Electrical and Computer Engineering, University of California Santa Cruz, Santa Cruz, CA 95064 USA

**Keywords:** Biotechnology, Electrical and electronic engineering

## Abstract

Continuous glucose monitoring from sweat and tears can improve the quality of life of diabetic patients and provide data for more accurate diagnosis and treatment. Current continuous glucose sensors use enzymes with a one-to-two week lifespan, which forces periodic replacement. Metal oxide sensors are an alternative to enzymatic sensors with a longer lifetime. However, metal oxide sensors do not operate in sweat and tears because they function at high pH (pH > 10), and sweat and tears are neutral (pH = 7). Here, we introduce a non-enzymatic metal oxide glucose sensor that functions in neutral fluids by electronically inducing a reversible and localized pH change. We demonstrate glucose monitoring at physiologically relevant levels in neutral fluids mimicking sweat, and wireless communication with a personal computer via an integrated circuit board.

## Introduction

Over 30.3 million people in the US have diabetes, a condition that now affects 18% of the worldwide population^1,2^. Typically, a person with diabetes has to monitor their blood sugar level (2–20 mM range) up to five times a day to regulate their metabolism. This monitoring involves the “prick test” to extract the blood, which could be painful, especially for children. Patients that avoid or forget to monitor themselves could suffer health repercussions. In severe cases, these repercussions can be fatal. Continuous glucose monitoring (CGM) that uses minimally invasive sources such as sweat is less demanding for patients. It improves healthcare by providing a higher data collection rate with an increased reliability while avoiding the discomfort of the “prick test^3^”. The glucose concentration in sweat ranges from 0.2 mM to 0.6 mM^4^, thus glucose sensing in sweat requires higher sensitivity than in blood. Devices capable of CGM are particularly useful and many sensors exist that can detect glucose from sweat and tears^5–9^. Google and Novartis have developed the smart Google contact lens, in which the sensing, storage, and transmission of the glucose levels occur on the contact lens^10^. In a parallel path, glucose monitoring skin patches are able to measure glucose in sweat^3^. All these examples are enzymatic sensors — the current standard for continuous monitoring of glucose^11^. These sensors detect the presence of glucose by measuring the rate of glucose oxidation from the enzymes glucose oxidase or glucose dehydrogenase^12^. For each glucose molecule oxidized, this reaction transfers an electron through a mediator to the sensing electrode. The sensing electrode records this electron transfer either by reading the electrode current or electrode potential. Enzymatic glucose sensors are highly sensitive, but the lifetime of these sensors is limited by decreasing enzymatic activity with time, this lifespan is typically one to two weeks. This relatively short lifetime increases cost and reduces the scope of enzymatic biosensors^11,13^.

Non-enzymatic glucose sensors based on metal oxides at the interface with nanostructured porous metals or carbon materials have longer lifetime than enzymatic sensors because they do not contain a biological component^14,15^. Metal oxide sensors detect glucose via the direct oxidation reaction of glucose with an activated metal oxide contact; the reaction results in an electron transfer to the contact which is recorded by the sensor as a current^16^. A highly sensitive and stable substrate for glucose detection is cobalt oxide at the interface with nanoporous gold^17^. However, this sensor only works in high pH (pH ≥ 11) because it requires the presence of hydroxide ions^18^. This restriction of working only at high pH is not limited to cobalt oxide sensors but applies to many metal oxide and inorganic material-based sensors that oxidize target molecules^19^. Thus, the development of metal oxide sensors for CGM applications has struggled since bodily fluids such as sweat and tears have a pH range of 4–7^20^. Here, we have developed a cobalt oxide-based glucose sensing platform that is able to detect glucose in solutions with the same pH as the bodily fluids such as sweat and tears. The proposed sensor includes the bioelectronic control of pH in the proximity of the cobalt-oxide sensor surface enabling sensing glucose at high pH even in an otherwise neutral fluid.

## Results

**Figure 1:**
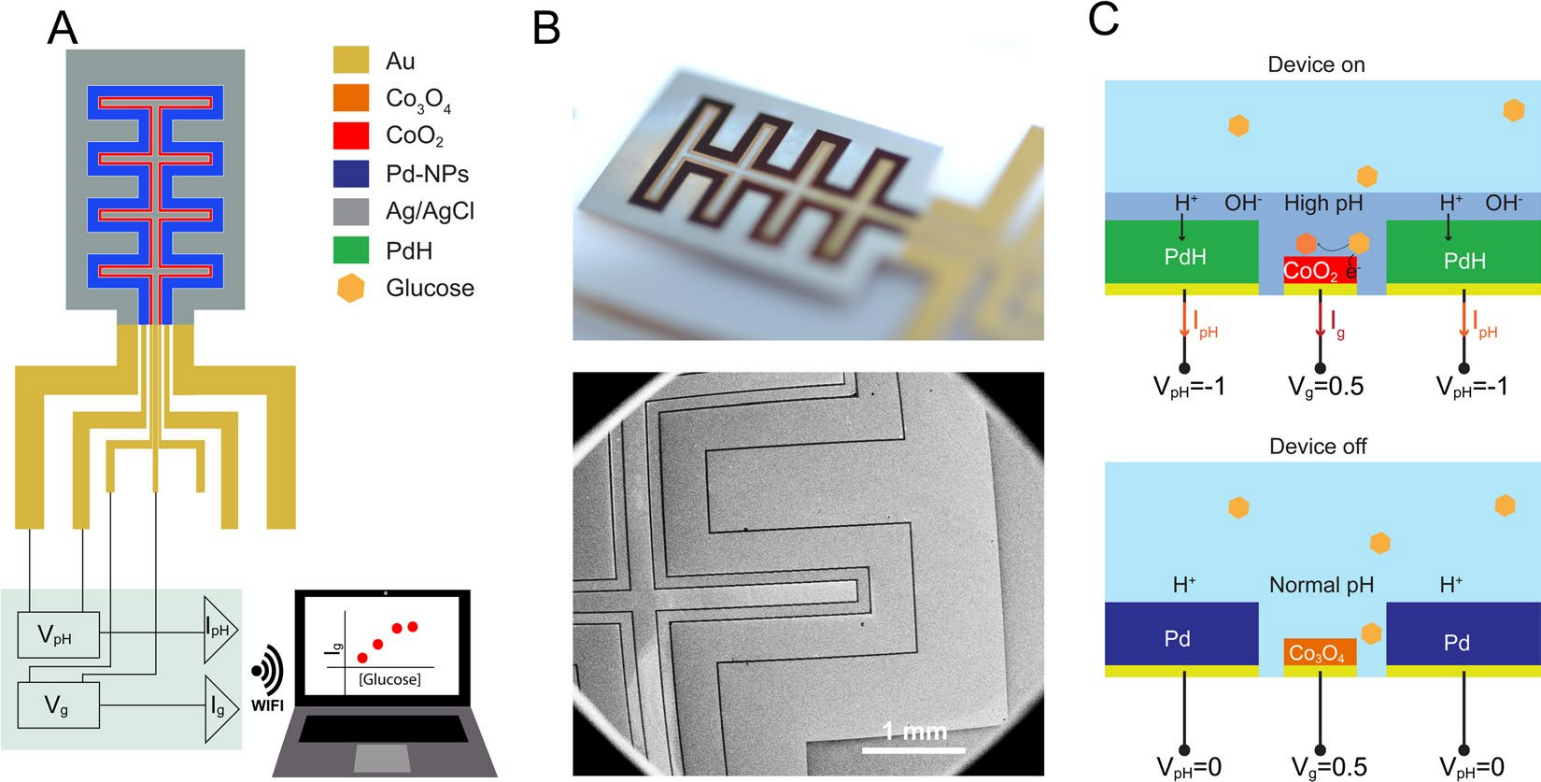
Non-enzymatic glucose sensor. (**A**) Schematic of the non-enzymatic glucose biosensor. A Pd contact (blue) locally creates basic conditions. In basic conditions, a nanoporous Au/Co_3_O_4_ (red) contact catalyzes glucose to gluconic acid. Two Ag/AgCl electrodes (grey) act as reference electrodes to the Pd and Au/Co_3_O_4_. A small conditioning board controls the device, acquires current, and transmits wirelessly to an external device. (**B**) Optical image (top) of the modified contacts glucose biosensor. Au contacts were modified with Pd, Co_3_O_4_, and Ag/AgCl by using electrodeposition. SEM image (bottom) of the platform showing the interdigitated contacts with a 20 μm gap between each contact. (**C**) Operating principle for glucose sensing. When the device is on (top), V_pH_ = −1 V, the Pd contact absorbs H^+^ from the solution and increases its pH. At high pH, the Au/Co_3_O_4_ contact is in its more reactive CoO_2_ oxidized state. With V_g_ = 0.5 V, the CoO_2_ contact oxidizes glucose and the resulting I_g_ is collected, which increases with increased glucose concentration. When the device is off (bottom), V_pH_ = 0 V, the pH is at physiological values, typically pH 7, no sensing occurs from the Au/Co_3_O_4_ and I_g_ = 0 A.

The glucose sensor comprises cobalt oxide (Co_3_O_4_), palladium (Pd), and silver/silver chloride (Ag/AgCl) contacts electrochemically grown on gold (Au) strips defined on a glass substrate. The Co_3_O_4_ contact is used to sense glucose, the Pd contact is used to change the local pH of the fluid, and the Ag/AgCl contacts act as reference electrodes that balance the half reactions for glucose and pH modulation respectively (Fig. 1A,B). These contacts are connected to an external circuit board that provides control voltages (V_g_ for the Co_3_O_4_ and V_pH_ for Pd), measures the contact currents (I_g_ for Co_3_O_4_ and I_pH_ for Pd), and provides signal analysis and wireless communication to a personal computer (Fig. 1A). The geometry of the platform consists of long and narrow interdigitated contacts with a gap of 20 μm. The specific geometry was designed with the aim to increase the I_g_ and I_pH_ currents by increasing the surface area and minimize the time for pH diffusion towards the Co_3_O_4_ by reducing the spacing between Pd and Co_3_O_4_ contacts. The novelty of this glucose sensing platform is the ability to create localized and transient alkaline conditions (high pH) for glucose sensing to occur on the Co_3_O_4_ contact even in neutral fluids (Fig. 1C). This transient high pH is needed because in neutral pH solution, at low voltage (e.g., V_g_ = 0.2–0.5 V), the glucose oxidation reaction does not occur on the Co_3_O_4_ contact and thus the presence of glucose cannot be detected. In order to induce local alkaline conditions of a solution starting at pH 7, we set V_pH_ = −1 V between the Pd and the Ag/AgCl. Pd has the ability to absorb H^+^ from the solution by first reducing H^+^ into H at the Pd/solution interface and then absorbing H into its metal lattice to form PdH_x_ with x being the atomic ratio of H to Pd and its value can reach up to 0.6^21^. In this fashion, our group has already demonstrated transfer of H^+^ to and from hydrated proton conducting polymers^22,23^ as well as membrane proteins^24,25^, modulation of solution pH for monitoring enzymatic reactions^26^, controlling bioluminescence^27^, and targeted cargo delivery to cells^28^. By setting the value of V_pH_, we control the pH of the fluid by multiple units in proximity to the cobalt oxide contact, thus creating a local environment for glucose sensing to occur. When the measurements are completed, the pH is restored to its original value, by switching the V_pH_ off which causes PdH_x_ to release its stored H back into solution as H^+^ (Fig. 1C).

**Figure 2:**
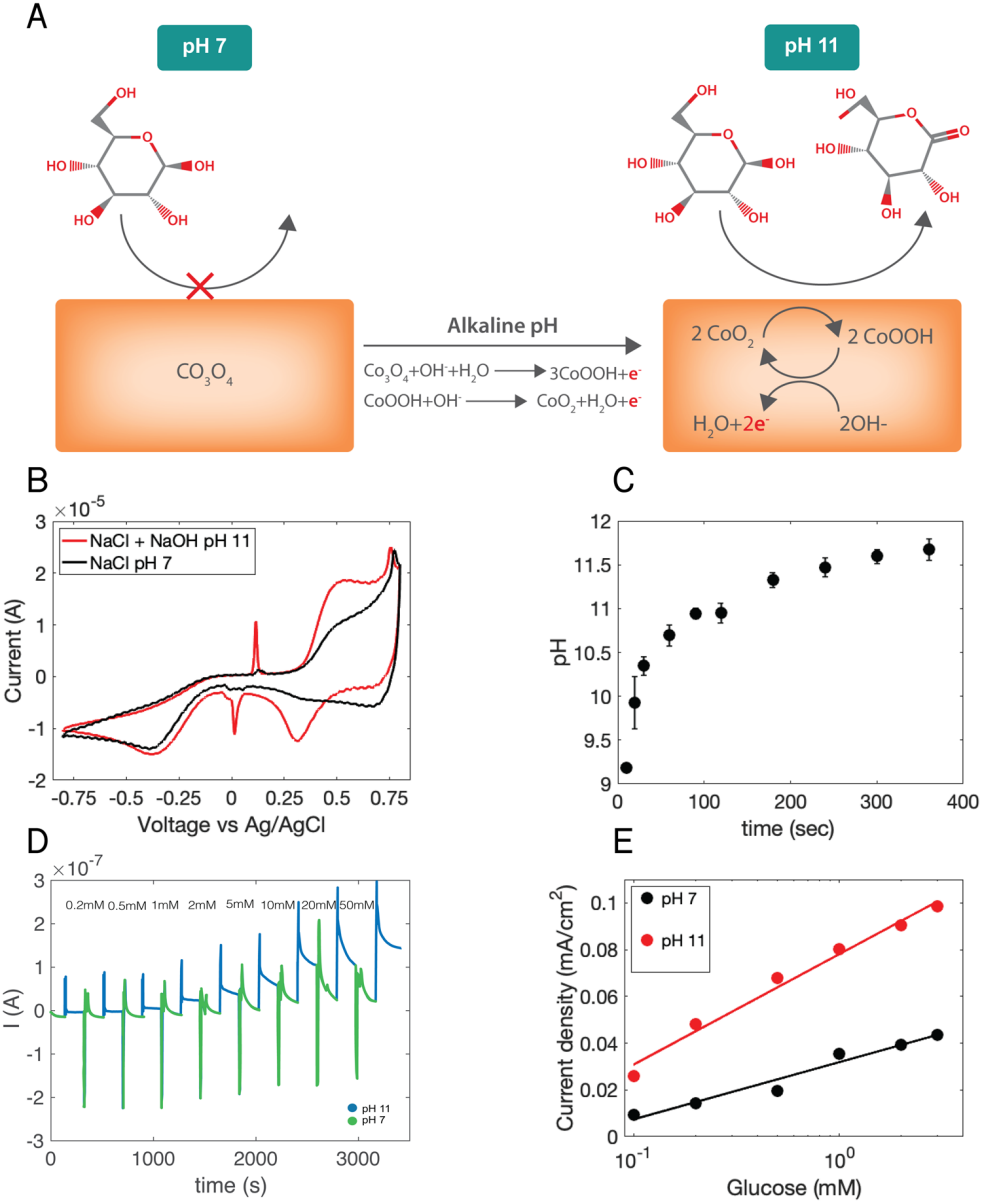
Glucose Sensor Operation. (**A**) Sensing mechanism of Co_3_O_4_ contacts. At pH 7, the contact is primarily Co_3_O_4_, which does not oxidize glucose. In alkaline conditions (pH ≥ 11), the contact is now mainly CoO_2_. CoO_2_ species react with glucose and are converted to CoOOH. This CoOOH is then oxidized back to CoO_2_. For every oxidized glucose molecule, the contact collects two electrons measured as I_g_. (**B**) Cyclic voltammetry of a nanoporous Au/Co_3_O_4_ contact in 0.1 M NaCl at pH 7 and 0.1 M NaCl + 0.001 M NaOH at pH 11 both containing 10 mM of glucose. (**C**) pH of 0.1 M NaCl (initial pH = 7) after pH modulation by a Pd electrode with a V_pH_ = −1 V between 10 s and 5 minutes. (**D**) Device operation. Current response of device at a constant voltage V_g_ = 0.5 V over increasing concentrations of glucose in 0.1 M NaCl solution. Pd contacts cycle the pH between pH 7 (V_pH_ ≥ 0 V) and pH 11 (V_pH_ = −1 V). During the pH 7 phase, glucose concentration is stepped. (**E**) Calibration curve of sensing platform versus glucose concentration with a fit for pH 11 and pH 7 induced by Pd for 100 sec.

The Au contacts are etched electrochemically by alloying/dealloying in the presence of zinc chloride following the protocol previously reported by Lang *et al*.^17^, to increase the surface area (Supplemental Figs 4 and 5) and improve the sensitivity towards glucose sensing. Cobalt oxide is then deposited on the gold surface as Co_3_O_4_ and, in solution, undergoes a series of oxidation reactions with hydroxide (Fig. 2A). These reactions increase the oxidation state from Co(II/III) to Co(III) and Co(IV), respectively^18^:

1 $$C{o}_{3}{O}_{4}+O{H}^{-}+{H}_{2}O\to 3CoOOH+{e}^{-}$$

and

2 $$CoOOH+O{H}^{-}\to Co{O}_{2}+{H}_{2}O+{e}^{-}$$

While both Co_3_O_4_ and CoOOH species can oxidize glucose, the primary mechanism for oxidation of glucose to gluconolactone in cobalt oxide sensors involves two Co(IV) atoms in the reaction^18,29^:

3 $$2Co{O}_{2}+{C}_{6}{H}_{12}{O}_{6}\to 2CoOOH+{C}_{6}{H}_{10}{O}_{6}$$

In (3), two Co(IV) atoms are reduced to Co(III) as CoOOH. These CoOOH species are in turn oxidized back to CoO_2_, for each Co(III) oxidized to Co(IV), two electrons are collected by the cobalt oxide contact and are recorded as current (I_g_) (2). At neutral pH, the concentration of hydroxide species necessary to create Co(IV) is low thus greatly limiting the glucose oxidation reaction kinetics. At high pH (Fig. 2A), the cobalt surface contains many more Co(IV) and the glucose oxidation reaction is faster allowing glucose detection at lower concentrations respect to neutral pH. For this reason, metal oxide sensors and other inorganic sensors that directly oxidize glucose operate at high pH^19^.

To see the behavior of the sensing element in our platform, we performed cyclic voltammetry measurements (CV) using a Co_3_O_4_ contact in NaCl solutions containing 10 mM glucose at pH 7 (Fig. 2B, black trace) and NaCl with 0.001 M NaOH at pH 11 (Fig. 2B, red trace). From the CV it is clear that the glucose oxidation reaction at pH 7 is barely detectable, while at pH 11 in 0.001 M NaOH the peak current associated with oxidation Co(III) to Co(IV) after the contact is reduced by the reaction with glucose occurs around V = 0.5 V (Supplemental Fig. 11). Similarly, we performed CV of glucose in pH 12 and pH 13. When the pH increases, both oxidation peak current magnitude increases as well as its peak position shifts to lower potential (Supplemental Fig. 10). In order to reproduce the alkaline conditions found when the glucose sensor is immersed in NaOH, we perform pH control in proximity to the cobalt oxide contact using the Pd contact with V_pH_ = −1 V for different amounts of time (t) in NaCl at pH 7 (Fig. 2C). For t < 300 s the pH increases with time because H^+^ are able to transfer from the solution to the Pd contact. However, at t = 300 s the solution pH saturates because there is a low concentration of H^+^ while an OH^−^ constant concentration is reached at the interface with the Pd. Even with lower V_pH_ = −3 V, the solution pH saturates at pH 11–12 (Supplemental Fig. 1). This transient pH change is reversible when the V_pH_ is returned to 0 V (Supplemental Fig. 2 and Video). We are limited in the magnitude of V_pH_ by electrolysis of water. We thus choose a V_pH_ ~ −1 V for our glucose sensing. This V_pH_ value corresponds to a solution of pH 11. For glucose detection, we measure the current at the cobalt oxide sensing contact (I_g_) with V_g_ = 0.5 V (Fig. 2D). However, the increased current from glucose oxidation can begin to occur in lower voltage V_g_ ~ 0.2 V (Supplemental Fig. 10). A low V_g_ ~ 0.2 V can be useful in order to improve the selectivity towards interferent species such as lactic acid and uric acid that exist in sweat. During the measurement we cycle the solution pH from neutral (V_pH_ = 0.3 V) to pH 11 (V_pH_ = −1 V). At neutral pH, I_g_ is very small for glucose concentrations below 1 mM. However, at pH 11, I_g_ raises above the noise level and further increases with addition of glucose in concentrations from 0.2 mM to 50 mM during periods of pH 11 (V_pH_ = −1 V). The resulting I_g_ is plotted against glucose concentration for 0.1–3 mM both at pH 7 and pH 11 (Fig. 2E). These data clearly show that the glucose oxidation is more efficient at pH 11 and is required for better sensitivity of our biosensor. Additionally, a Langmuir isotherm fit corresponding to the adsorption of glucose at the surface of the Co_3_O_4_ is in agreement with the experimental data at the concentration range between 100 μM–3 mM. allowing for detection of glucose in sweat concentrations while electronically inducing basic conditions in electrolytes of physiological pH. Co_3_O_4_ sensors have shown limit of detection in the nanomolar range in high alkaline conditions^18^, however, in this work we did not perform measurements for finding the detection limit. At pH 11, the reaction rate, is higher because there are more Co (IV) species available to oxidize the glucose. It is worth mentioning that when V_pH_ goes from V_pH_ = −1 V to V_pH_ ≥ 0 the pH in the vicinity of the Co_3_O_4_ is higher than pH 7 due to slow H^+^ diffusion. This may result in the increased signal in the black curve of Fig. 2E (pH 7).

**Figure 3:**
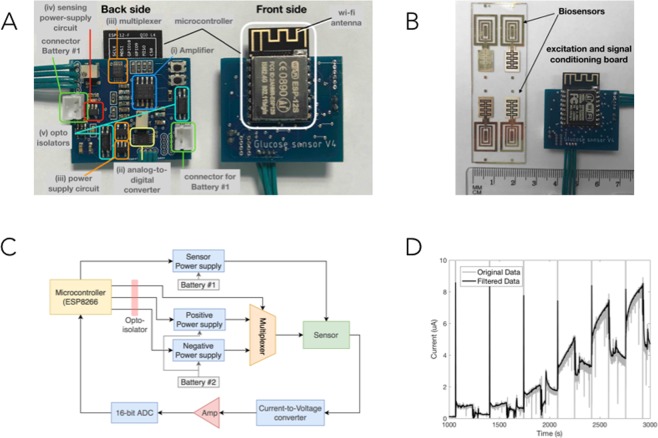
Integrated biosensor and sensing/excitation board. (**A**) Photo of the excitation and signal conditioning board used to provide V_pH_ and V_g_ and measure the current generated by the chemical reaction to determine the glucose level (I_g_). (**B**) The biosensor (left) and the microcontroller signal conditioning/sensing board (right) that applies V_pH_ and V_g_, extracts and amplifies the sensing current, and transmits the results via WI-FI for further analysis. Please note that one microscope slide fits 4 experimental biosensors. (**C**) The circuit schematic explaining how the components shown in (**A**) are integrated with the biosensor. (**D**) I_g_ vs time recording with microcontroller from sensing at 0 mM, 0.1 mM, 0.2 mM, 0.5 mM, 1 mM, 2 mM, 3 mM glucose concentration in 0.1 M NaCl solution.

To demonstrate the feasibility of the non-enzymatic cobalt oxide glucose sensor in real world continuous glucose monitoring measurements, we developed a prototype low-cost and low-power miniature board that can apply voltages (V_g_, V_pH_), record and condition the signal as well as transmit it to an external device (WI-FI) for storage and post processing (Fig. 3A). The board includes a WI-FI enabled microcontroller, a multiplexer, an amplifier, and analogue to digital converter, and two batteries. We connect the board to the Co_3_O_4_, Pd, and Ag/AgCl contacts to the inputs and outputs of the board (Fig. 3B). A high-level schematic is presented in Fig. 3C and a more detailed one is presented in the Supplemental Fig. 3. The board features two electronic circuits, which are isolated from each other and each of them has an adjustable power circuit. The first circuit supplies V_g_ (from 10 mV to 3.2 V) to the Co_3_O_4_ contact for glucose sensing to occur, and the second circuit supplies V_pH_ (from −1.1 V to 0.3 V) to the Pd in order to create the alkaline conditions. We used a WI-FI enabled microcontroller (ESP8266,) and a 16-bit analog-to-digital converter to measure I_g_. Figure 3D shows the experimental I_g_ current acquired from the glucose sensing platform and the circuit board by increasing concentrations of glucose in a NaCl solution made to reproduce human sweat. This I_g_ data is filtered by the conditioning board to remove noise and recorded with a customized software in a personal laptop wirelessly connected to the microcontroller. Figure 3D shows the measured current as a function of time in seconds (gray) as well as the filtered signal using a moving average filtering technique with a 1/3 of a second window (black). The data is consistent with what we acquired with the bulkier and non-portable potentiostat and semiconductor parameter analyzer and follows the analogous calibration curve presented in Fig. 2E.

## Conclusions

We have developed a non-enzymatic metal oxide glucose sensor that is able to detect physiologically relevant glucose levels in neutral bodily fluids such as sweat and tears. This sensor is superior to other metal oxide glucose sensors because it does not require an alkaline fluid for operation. To sense glucose in neutral fluids, this sensor induces a localized and reversible pH change with a Pd contact that absorbs H^+^ from the neutral fluid and increases the pH. This flexibility allows for the seamless integration with current glucose sensing platforms such as contact lenses^10^, and skin patches^3^. With respect to the current enzymatic sensors, this metal oxide sensor does not suffer from limited lifetime due to enzyme degradation over time. This strategy of controlling local pH to enable sensing in neutral biological fluid is broadly applicable to other metal oxide and oxidative inorganic sensors for biologically relevant analytes including but not limited to ascorbic acid, dopamine, glycerol, ethylene glycol, and nitrite^30^.

## Materials and Methods

### Glucose sensor fabrication

Glass slides were sonicated for 20 min in 80% v/v acetone and 20% v/v iso-propanol (IPA), and dried with N2. S1813 (Dow chemicals) photoresist was deposited on top of the glass substrates, following standard protocols (Spin-coated at 3000 RPM, baked 1 min. at 110 °C), to create the Au patterns. A 5 nm Titanium adhesion layer and a 120 nm Au layer were evaporated on glass microscope slides. Deposition of photoresist was repeated prior to each electrodeposition following the same process. To increase the sensitivity of the sensor, we increased surface area of Au strip a process adapted from Lang *et al*. (Supplemental Fig. 4)^17^. Nanoporous Au was produced by electrochemically etching the 100 nm thick Au layer with a solution of 1.5 M ZnCl_2_ in Benzyl Alcohol at 120 °C. To cycles of a cyclic voltammetry routine from −0.4 V to 1.7 V vs AgCl was performed with Zn wire reference and counter electrodes and a Metrohn Autolab Potentiostat (PGSTAT128N). This routine corresponds to two rounds of Zn-Au alloying/dealloying. The devices were then washed with 0.1 M H_2_SO_4_, IPA, and di-water. This treatment was only performed on the contact destined for cobalt oxide. The nanoporous Au has higher capacitance resulting from a larger surface area (Supplemental Fig. 5). Cobalt Oxide was deposited from a solution of 5 mM Cobalt(II) Nitrate suspended in 0.1 M H_2_SO_4_. A CV routine (−1.2 V to −0.2 V) with a glass AgCl reference electrode and a Pt wire counter electrode. This was performed with a Metrohn Autolab Potentiostat (PGSTAT128N). To deposit Pd, we used 10 wt.% Palladium Nitrate (PdNO_3_), purchased from sigma, and diluted with di-water to give a 1 wt.% PdNO_3_ solution. PdNPs were electrochemically deposited onto the Pd contacts using a DC voltage of V = −0.3 V with a deposition time of 3 seconds with a glass AgCl reference and Pt counter electrode (Supplemental Fig. 6). This resulted in a darkening of the contacts where the NPs were successfully deposited. The Pd nanoparticles have an increased surface area which greatly enhances the pH change effect over planar Pd and area stable over many pH cycles (Supplemental Figs 7 and 8). To create the Ag/AgCl electrode, we electrodeposited Ag on top of the Au contact in the reference electrodes by using a constant current of 0.5 mA for 15 s (outer contact) and 0.15 mA for 150 s (inner contact), from a solution containing 50 mM of AgNO_3_ and 0.2 M sulfuric acid in di-water. A glass Ag/AgCl (V ~ 0.2 vs SHE) electrode was used as a reference electrode and Pt wire was used as a counter electrode. CV was used to form AgCl on the Ag, by using a solution containing 0.5 M NaCl in di-water. 5 cycles were carried from −0.5 to 0.9 V with a scan rate of 50 mV/s (Supplemental Fig. 6). An SU8 photoresist layer was patterned to insulate the Au interconnect and define the area of the electrodes.

### Characterization of cobalt oxide contact

The planar Au, nanopourous-etched Au, and nanoporous Au/Co_3_O_4_ coated surfaces were characterized by cyclic voltammetry in 0.1 M NaCl solution (pH 7) vs a glass AgCl electrode (Supplemental Fig. 9). A CV current typical gold profile (black) is amplified when the surface area of the contact is increased after etching (blue). After deposition of Co_3_O_4_, the recorded current is lower than that of the nanoporous Au as a result of the oxide film being more resistive, this is true until a broad peak at 0.5 V which is characteristic of further oxidation of Co_3_O_4_.

### pH control and characterization

pH cycling was controlled with an Autolab potentiostat connected to the Pd contact and an external AgCl pellet (electrode potential value V = 0.21 vs standard hydrogen electrode). The quantification of solution pH was recorded with a micro-pH meter (Fisher Scientific). Diffusion of pH in the Co_3_O_4_ was recorded with a Keyence VHX-5000 series digital microscope. The solution was 0.1 M NaCl initially at pH 7 with a universal pH indicator dye (Fisher Chemical) at a volume of 0.1 mL.

### Glucose measurements - NI and potentiostat

pH cycling was controlled by an Autolab potentiostat connected to the on-chip Pd and AgCl electrodes. The on-chip cobalt oxide-AgCl circuit was controlled with an NI PXI with a digital multimeter and source measuring unit. Measurements began initially in 0.1 M NaCl in di-water. During cycling of pH, glucose concentration was increased during periods of pH 7.

### Glucose measurements - microchip

The board consists of two layers (sides). The WI-FI-enabled microcontroller (Espressif Systems microcontroller ESP8266) is installed on the “front” side while the rest of the electronics on the “back” side. This include: (i) an analog amplifier INA122U Texas Instruments, (ii) a 16-bit analog-to-digital converter (ADS1115 - Texas Instruments), (iii) a multiplexer (Texas Instruments TS5a4624) which together with a fixed +1.4 V power supply circuit (ABLIC S-13R1A14) and an adjustable (+0.1 to +3.2 V) adjustable power supply circuit (Microchip MCP601OT) we obtained the voltages required for the excitation (+0.3 V and −1.1 V), (iv) a power supply (Microchip MCP601OT) for the sensing circuit, (v) two opto-isolator components (Vishay Semiconductor VOS618A) to separate the sensing and excitation sides of the circuit board. Measurements were performed between the on-chip Pd contact and an external AgCl pellet and the on-chip Co_3_O_4_ contact and an external AgCl pellet. Measurements began initially in 0.1 M NaCl, glucose concentration was increased during periods of pH 7.

### Electrical characterization (CV)

Device characterization was done utilizing both an Autolab potentiostat and national instruments (NI) PXI with a digital multimeter (DMM) and a source measurement unit (SMU). A custom labview program was controlling the NI system. Potentiostat tests were run to gauge the performance of the devices, cyclic voltammetry, and frequency response analysis (FRA).

## Supplementary information

Supplementary Information
