# Supplementary material for: A non-enzymatic glucose sensor enabled by bioelectronic pH control
Source: Sci Rep. 2019 Jul 26;9:10844. doi: 10.1038/s41598-019-46302-9 (PMC6659689; doi:10.1038/s41598-019-46302-9)
Supplement: Supplementary file 1 — Supplementary Information [file 41598_2019_46302_MOESM1_ESM.docx]

**­­­­A non-enzymatic glucose sensor enabled by bioelectronic pH control**

**Authors**

Xenofon Strakosas^†^ and John Selberg^†,^ Pattawong Pansodtee, Nebyu Yonas, Pattawut Manapongpun, Mircea Teodorescu, and Marco Rolandi^1*^

**Affiliations**

^1^Department of Electrical and Computer Engineering, University of California Santa Cruz, Santa Cruz, CA 95064. *E-mail: mrolandi@ucsc.edu

^†^These authors contributed equally to this work.

**Supplementary Materials**

**­­**

**Supp Fig. 1:** pH of 0.1ml volume of 0.1M NaCl solution after 120s of pH change versus applied V_pH_.

**Supp Fig 2:** pH changes detected optically in NaCl with pH indicator solution. The palladium contact (black) cycled between -1V and +0.3V vs an AgCl pellet electrode for 1 minute at each voltage. The solution switched between an initial neutral solution (yellow) at t=0s (left), to basic pH (blue) at t=60s after 1 minute of -1V on the Pd (second from left). The cycle was repeated neutral conditions at t=120s after +0.3V on the Pd, and basic conditions at t=180s. Scale = 0.4mm.


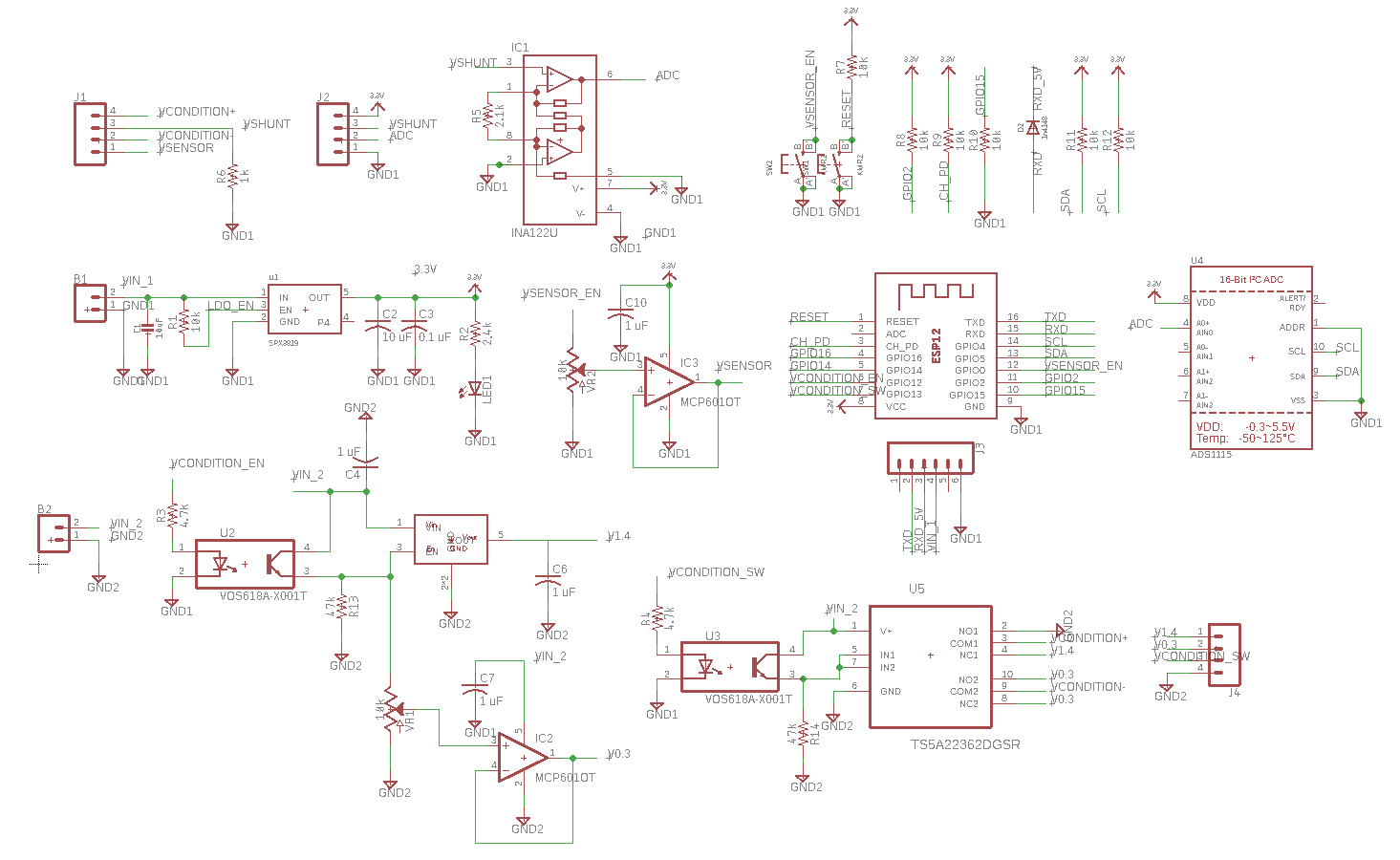


**Supp Fig 3:** PCB schematic. Sensor unit PSU is generated from voltage divider on a high precision potentiometer (VR2) which later buffers through unify gain op-amp. R6, resistor is connected in-line with the sensor unit, its work as shunt resistor that converts current to voltage. The voltage across R6 is amplified with a gain of 100 by INA122U, instrument amplifier which latter sense by ADS1115, 16-bits analog to digital converter. Similar to the sensor unit PSU, part of pH PSU use potentiometer (VR1) and unify gain op-amp to create one of the signals. The second signal is created from a 1.4 V voltage regulator. Down the line, the two signals are multiplexed through TS5A22362DGSR, analog multiplexer for pH PSU output. Both PSU can be enabled/disabled by the microcontroller. Moreover, pH PSU is isolated from the microcontroller due to different battery and VOS618A, opto-isolator.

**
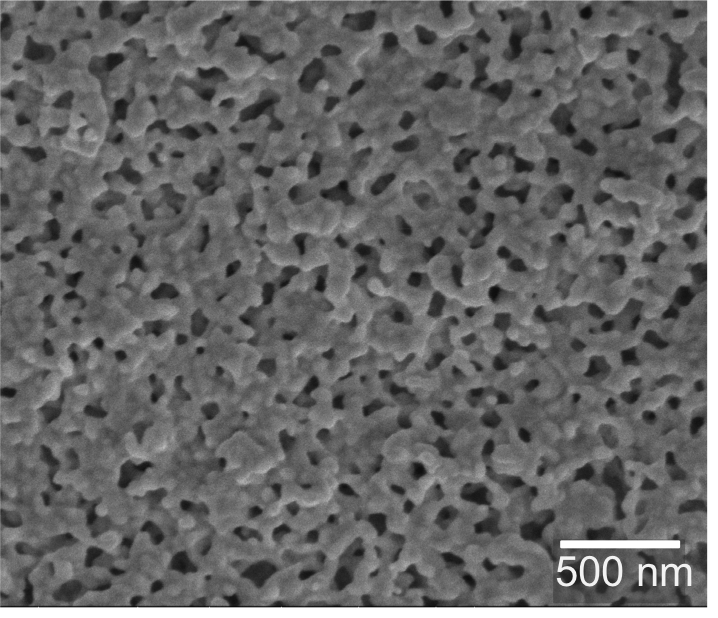
**

**
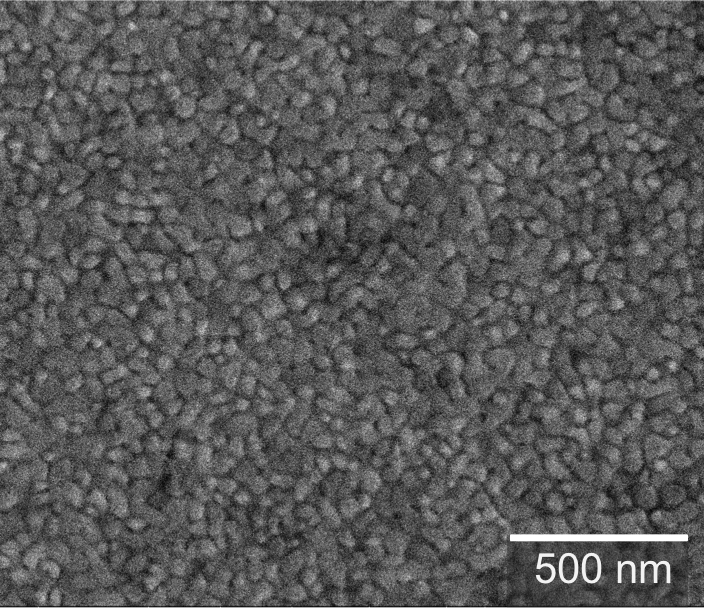
**

**Supp Fig 4:** Au Etch SEM. Un-etched Au (left). Nanoporous Au etch with ZnCl_2_ in Benzyl Alcohol at 120C (right).

**Supp Fig 5:** Bare Au vs nanoporous Au Nyquist plot generated from impedance spectroscopy measurements showing that the nanoporous Au has a higher capacitance evident by the larger value of the imaginary impedance.

**
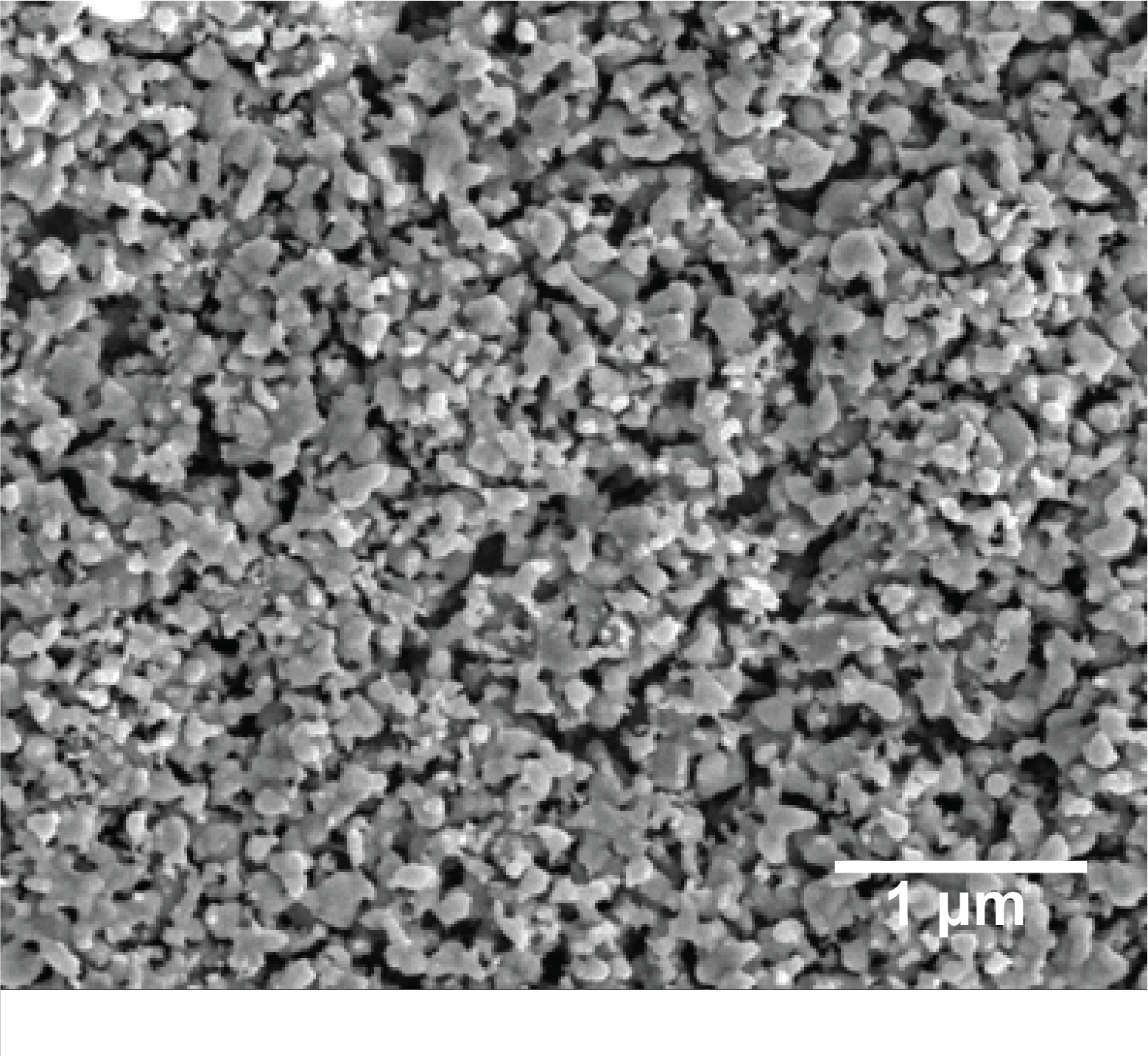

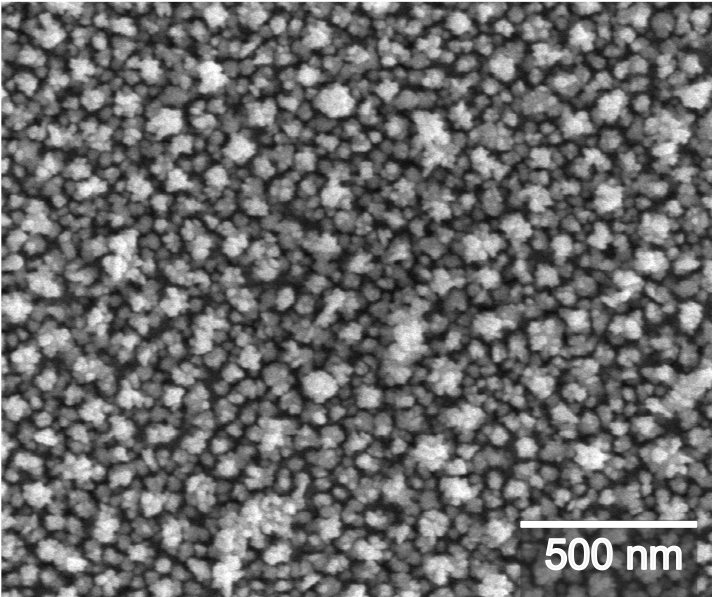
**­­­­

**Supp Fig 6:** SEM­ Pd Nanoparticles­ (left). Ag/AgCl nanoparticles

**
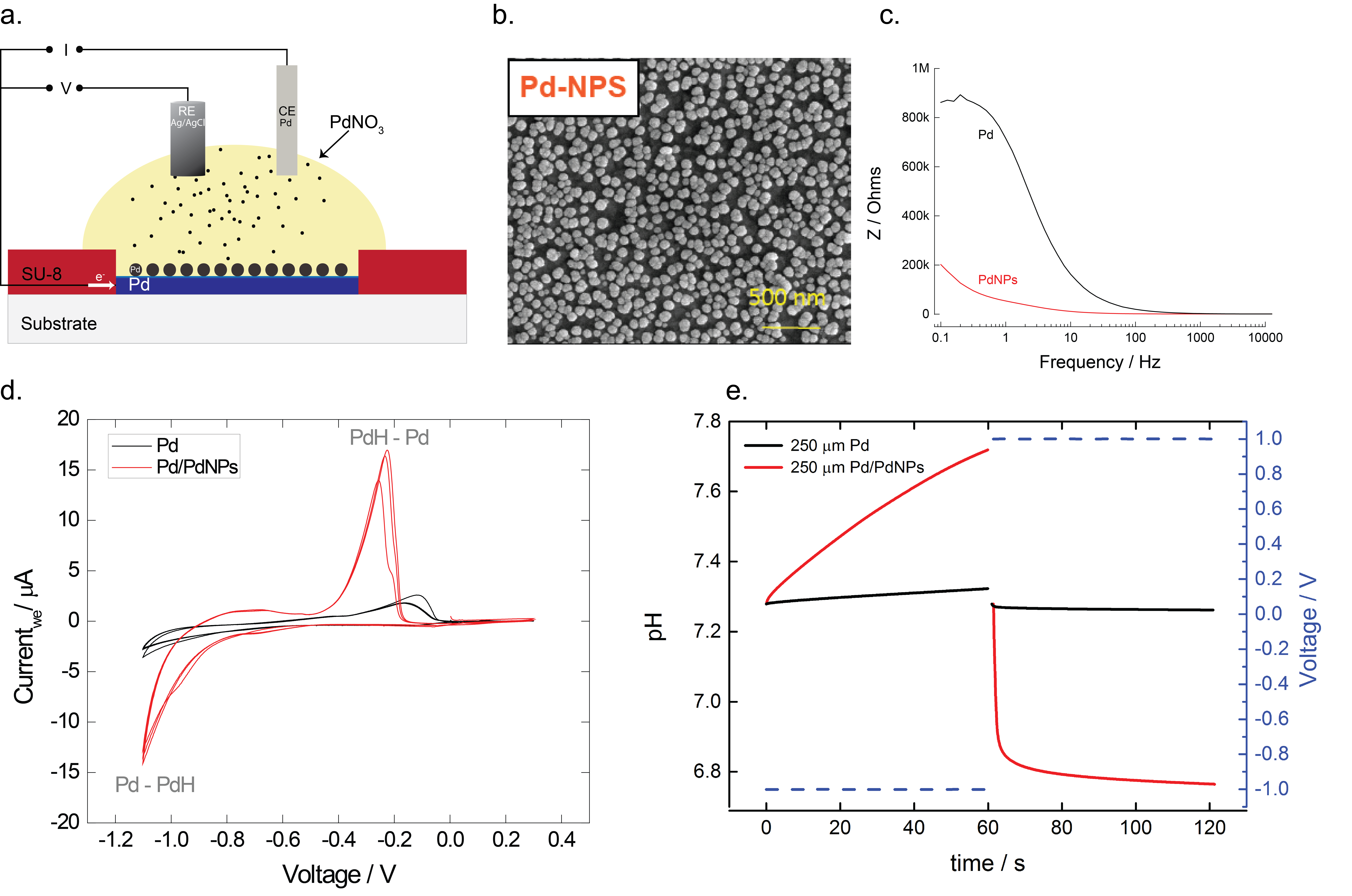
**

**Supp Fig 7.** Cyclic voltammetry of a Pd contact versus Pd nanoparticles contact (250x250 μm) showing the increased performance of electrodeposited Pd. The current which corresponds to the H^+^ transfer between solution and Pd is higher for electrodeposited Pd

**
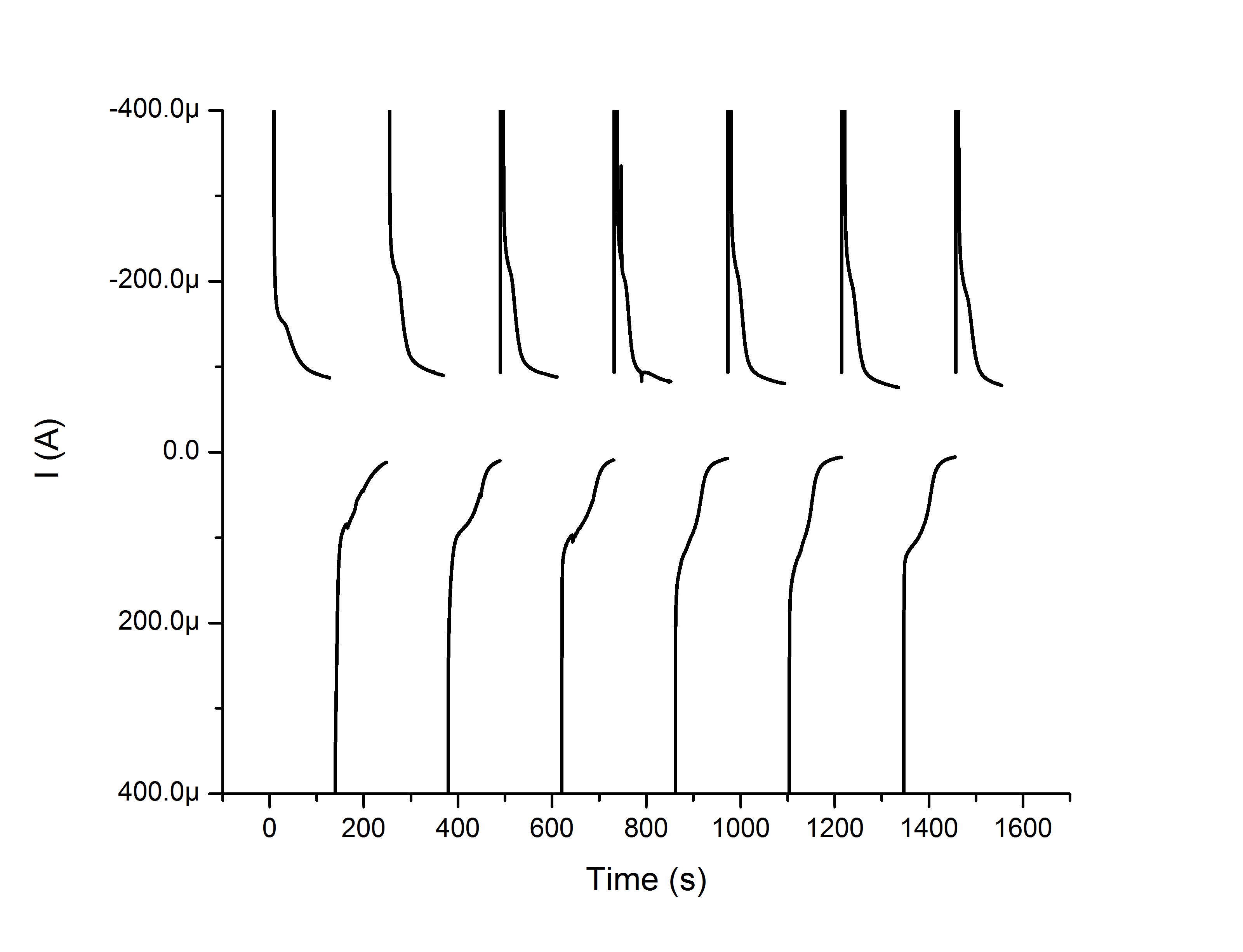
**

**Supp Fig 8.** Current recording from Pd contact during pH cycling. The Pd contact was switched between -1V and +0.3V vs AgCl. The current is reproducible for multiple cycles.

**
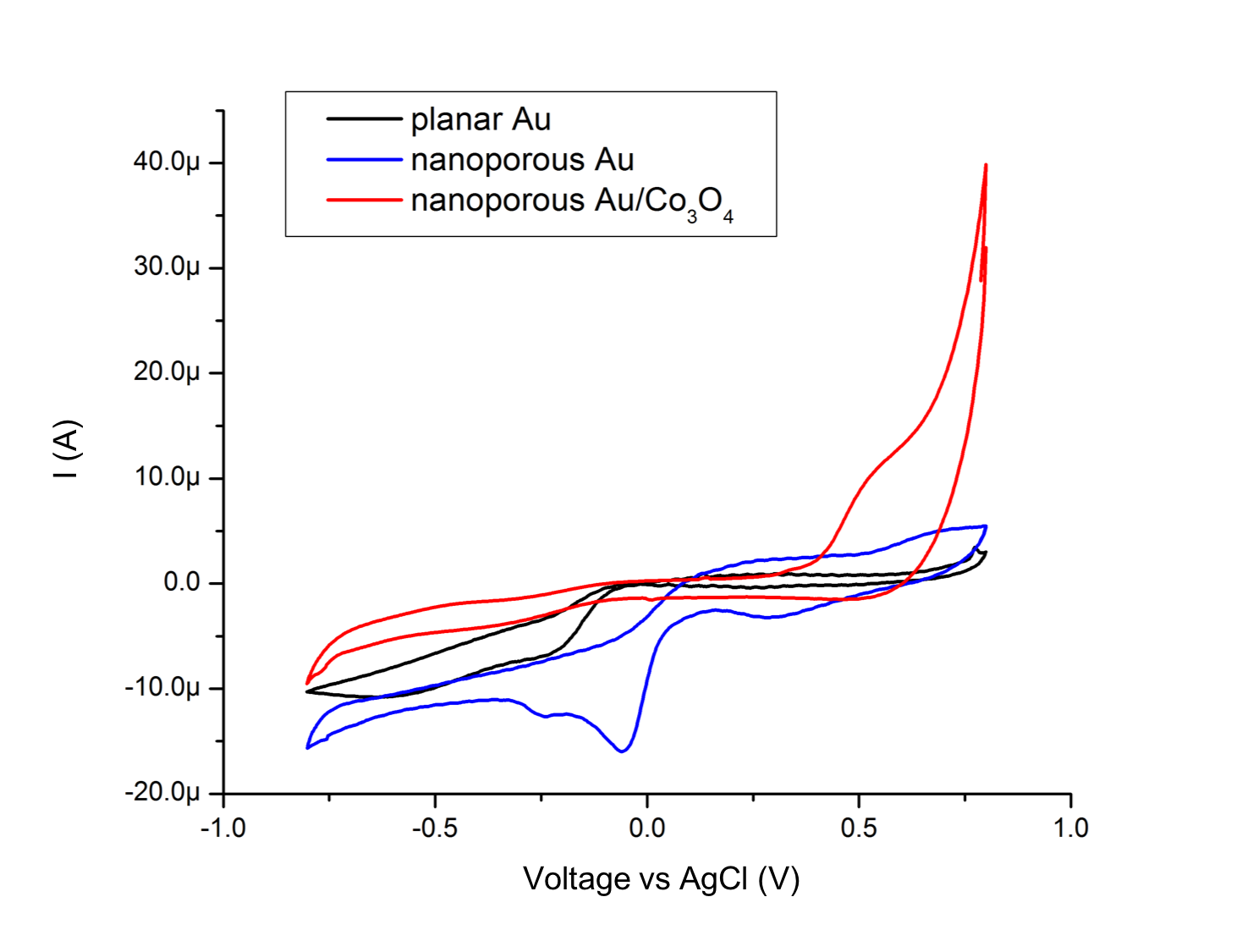
**

**Supp Fig 9.** Cyclic voltammetry of planar Au, nanoporous Au, and nanoporous Au/Co_3_O_4_ in 0.1M NaCl in the absence of glucose.

**Supp Fig 10.** Cyclic voltammetry of nanoporous Au/Co_3_O_4_ in 0.1M NaCl pH 7 and 0.1M NaCl with 0.001M NaOH (pH 11), 0.01M NaOH (pH 12), and 0.1M NaCl with 0.1M NaOH (pH 13) in the presence of 10mM glucose.


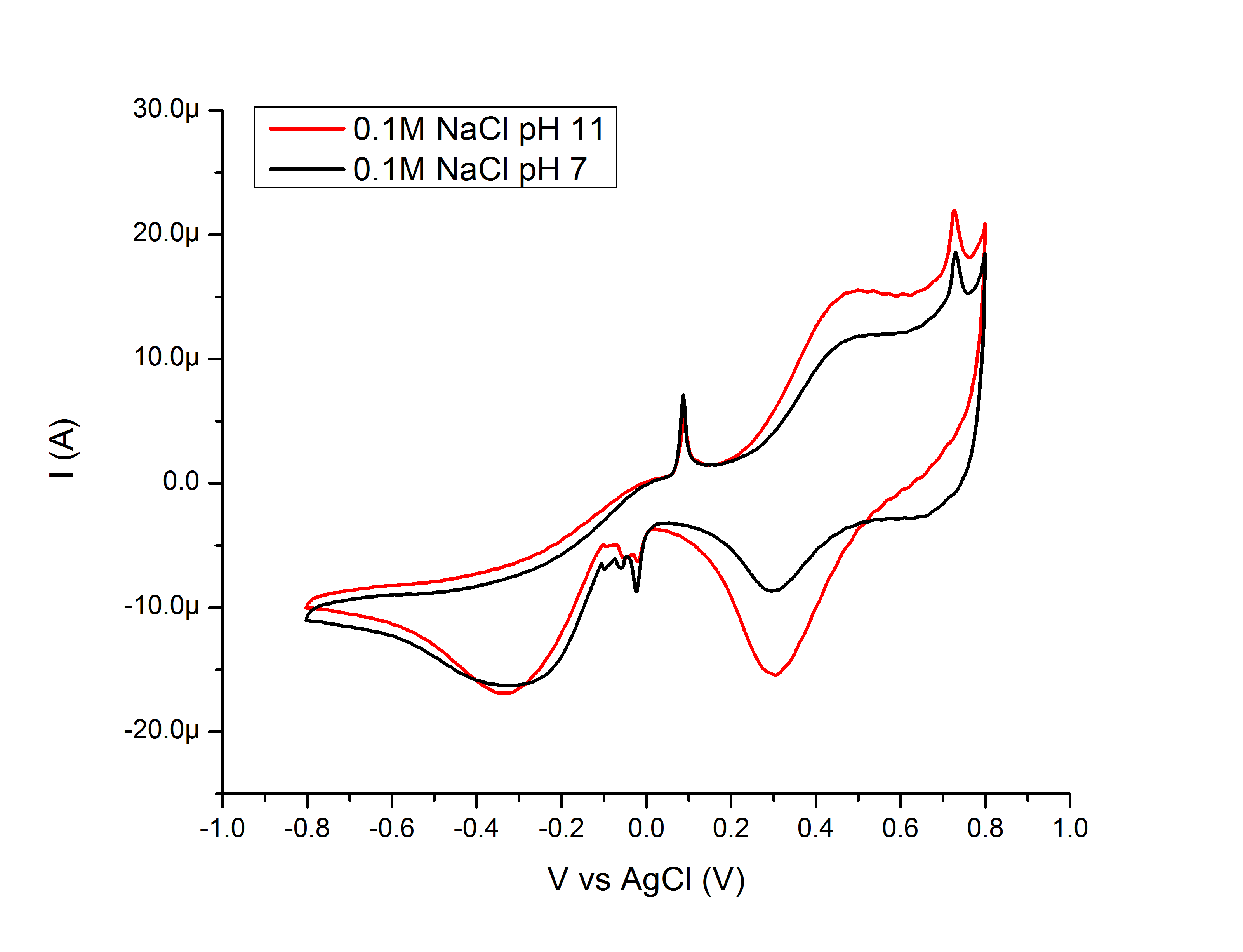


**Supp Fig 11.** Cyclic voltammetry of nanoporous Au/Co_3_O_4_ in 0.1M NaCl pH 7 and 0.1M NaCl with 0.001M NaOH (pH 11),
